# Glaucoma awareness among people attending ophthalmic outreach services in Southwestern Ethiopia

**DOI:** 10.1186/1471-2415-10-17

**Published:** 2010-05-28

**Authors:** Addis Tenkir, Berhan Solomon, Amare Deribew

**Affiliations:** 1 Department of Ophthalmology, Collage of Public Health and Medical Sciences, Jimma University, Jimma, Ethiopia; 2 Department of Epidemiology and Biostatistics, Collage of Public Health and Medical Sciences, Jimma University, Jimma, Ethiopia

## Abstract

**Background:**

Individuals may need to be aware of glaucoma and seek assessment regularly in order to diagnose the condition early. Awareness of glaucoma has not been previously documented in an Ethiopian setting.

**Objective:**

The main aim of this study was to assess the level of awareness of glaucoma among people attending outreach ophthalmic services.

**Methods:**

We conducted a cross-sectional survey in April 2009 of all people 40 years of age or older who presented during a two-week outreach service period in Agaro town, southwestern Ethiopia. Data on demographics and awareness of glaucoma were collected through face to face interview using a pretested structured questionnaire.

**Results:**

340 people participated in the study. Their mean age was 54.5 (SD 10.6) years. About 43% were illiterates and 37.6% were farmers. Only eight people (2.4%) were aware of glaucoma. The association between awareness and attaining high school or better education was statistically significant (p < 0.0001).

**Conclusion:**

Awareness of glaucoma in this population is very low. An efficient information, education and communication (IEC) strategy needs to be designed to increase knowledge of the community about glaucoma so that early diagnosis and treatment of individuals with this condition may be possible.

## Background

Glaucoma is the second leading cause of blindness worldwide [1]. Up to 50% of glaucoma patients are already blind in one eye at presentation in Africa including Ethiopia [2]. Eye health education that influences people to participate in regular ophthalmologic care may be an important step to detect glaucoma early, thereby preventing needless blindness. Not only can education and preventive eye care save needless suffering, it can also reduce the economic burden of the disease [3]. Subgroups of the population who are at highest risk both for developing the disease and having insufficient knowledge about it need to be identified and targeted in order to most effectively use resources for public education.

The Department of Ophthalmology of Jimma University provides outreach ophthalmic activities in addition to regular services at its tertiary eye unit. The outreach activities include general eye health evaluation, refraction, trichiasis surgery and cataract surgery. These outreach services are conducted at ten sites located around Jimma town in southwestern Ethiopia. A single outreach program usually lasts for about two weeks. Each site is visited at least once a year. A wide range of individuals from different socioeconomic and educational background present for eye evaluation during outreach programs. To our knowledge, based on a search of internet-based databases, this is the first study done to assess awareness of glaucoma in an Ethiopian setting.

## Methods

We conducted a cross-sectional survey during a two-week ophthalmic outreach campaign in April 2009 in Agaro town. It is located 390 kilometers southwest of Addis Ababa, the Ethiopian capital city. Ethical clearance was granted by the Ethical Review Board of the Collage of Public Health and Medical Sciences of Jimma University. Free and informed consent was obtained from each participant.

Agaro town is the administrative capital of Gomma Woreda (*district*), one of the thirteen Woredas in Jimma Zone (*province*). The zone is one of the twelve zones in Oromia Regional State. Ethiopia has a federal system of government consisting of nine regional states and two city administrations. Based on figures published by the Central Statistical Agency in 2008, Gomma woreda has an estimated total population of 216,662, of whom 110,488 (51.0%) are men and 106,174 (49.0%) are women; 14,262 (6.6%) of its population are urban dwellers, which is greater that the Zone average of 5.7% [4]. There are one health center, 18 clinics and 16 rural drug vendors with 10 beds, 2 doctors, 6 nurses, 3 technicians and 26 health assistants in the woreda. Neither a resident ophthalmologist nor an ophthalmic nurse is available in Agaro town [5].

A total of 340 people aged 40 years or older presented for eye evaluation during the two-week period and all constituted the study population.

Data on demographics and awareness of glaucoma were collected by trained ophthalmic nurses through face to face interview using a structured questionnaire. The questionnaire was initially prepared in English. It was translated to Amharic and Affan Oromo. Back translation was made by independent individuals to check for consistency in meaning. The interview was conducted in the language each participant understood best.

The questionnaire was pretested on 50 randomly selected people 40 years of age or older who presented during a previous outreach campaign in Nada Woreda in the same Zone. We made necessary modifications and clarification of terms based on the pretest procedure. The questionnaire was designed to be brief and easily understandable. Most of the questions were close-ended. Respondents were not prompted to possible responses. Data were recorded as part of the initial evaluation of each patient seeking treatment during the campaign.

Questions regarding awareness of glaucoma were asked after collecting demographic information. Respondents were first asked whether or not they heard about glaucoma and where their source of information was. They were enquired to describe what they understood by glaucoma and their responses were recorded in their own words. A participant was classified as being aware of glaucoma if s/he said 'yes' to the question 'have you heard about glaucoma?' and gave such answers as 'glaucoma is high eye pressure', 'glaucoma is high eye pressure causing blindness', 'glaucoma causes damage to the eye nerve' or similar answers when asked 'what do you understand by glaucoma?'. Those who were aware of glaucoma were then asked whether they knew risk factors for glaucoma. We also asked whether they knew anyone who lost sight due to glaucoma, and whether one can have glaucoma without having any symptoms. Participants were also asked about personal and family history of glaucoma, history of eye surgery, previous screening and perceived risk of developing glaucoma.

Statistical analyses were performed with SPSS 16.0 (SPSS Inc, Chicago, Illinois) software. For the purpose of statistical analysis, we classified "educational level" as high school or better (those who completed grades 9 to 12 or attained better) and less than high school. The presence of associations between awareness of glaucoma and demographic attributes such as age, sex, and educational level were tested using the univariate Fisher exact test, Chi-square test or Student's t-test. All p-values reported are two-tailed and significance was defined as p < 0.05.

## Results

A total of 340 subjects were interviewed during the two-week study period. The age of participants ranged between 40 and 88 years with mean age of 54.5(SD 10.6) years. Majority of them were males (69.7%), married (95%), and Muslims (59.7%). About 43% were illiterates and 37.6% were farmers (Table 1).

**Table 1:** Sociodemographic characteristics of respondents at an ophthalmic outreach site in southwestern Ethiopia, 2009.

Sociodemographic characteristics	no (%) [N = 340]
**Age group (years)**	
40-49	120(35.3)
50-59	105(30.9)
60-69	76(22.3)
70-79	32(9.4)
≥80	7(2.1)
**Gender**	
Male	237 (69.7)
Female	103 (30.3)
**Marital Status**	
Married	323(95.0)
Single	9(2.6)
Widow	8(2.4)
**Religion**	
Muslim	203(59.7)
Christian	137(40.3)
**Education**	
Illiterate	146(42.9)
Read and write only	67(19.7)
1-4 grade completed	31(9.1)
5-8 grade completed	39(11.5)
9-12 grade completed	30(8.8)
Collage education	27(8.0)
**Occupation**	
Farmer	128(37.6)
Housewife	74(21.8)
Govt/nongov't employee^a^	68(20.0)
Merchant	32(9.4)
Daily laborer	13(3.8)
None/dependent on family	19(5.6)
Others^b^	6(1.8)

^a^employee of a governmental or non-governmental organization

^b^Priest/Imam (3), Pensioner (3)

Only 8 (2.4%) of them had some understanding about glaucoma (Table 2). The difference in awareness of glaucoma in relation to age, gender, marital status or religion was not statistically significant (Table 3). Glaucoma awareness was positively associated with attaining educational level of high school or better (p < 0.0001 (Table 3).

**Table 2:** Awareness of glaucoma in relation to sociodemographic characteristics of respondents at an ophthalmic outreach site in southwestern Ethiopia, 2009.

Characteristics [N = 340]	Aware [n = 8]	Not aware [n = 332]
**Gender**		
Male	5	232
Female	3	100
**Age**		
40-49	4	116
50-59	4	101
60-69	0	76
70-79	0	32
80+	0	7
**Marital status**		
Married	8	315
Non-married	0	9
**Religion**		
Muslim	4	199
Christian	4	133
**Education**		
Illiterate	0	146
Read & write only	1	66
1-4 grade completed	0	31
5-8 grade completed	0	39
9-12 grade completed	2	28
Collage education	5	22
**Occupation**		
Farmer	1	127
Housewife	1	73
Employee	6	62
Merchant	0	32
Daily laborer	0	13
Others	0	25

**Table 3:** Predictors of awareness of glaucoma among people attending ophthalmic outreach services in southwestern Ethiopia.

Characteristics [N = 340]	Aware [n = 8]	Not aware [n = 332]	p-value
**Gender**			
Male	5	232	.702
Female	3	100	
**Mean age (SD)**	49.25 (6.84)	54.65 (10.62)	.154
			
**Education**			
High school or better	7	50	<.0001
Less than high school	1	282	

The remaining 332 (97.6%) participants were not aware of glaucoma. The average age of those who were unaware of glaucoma (54.65 ± 10.62) was slightly higher compared to those who were aware (49.25 ± 6.84) although the difference was not statistically significant (P > 0.1) (Table 3).

The responses of those study participants who were aware of glaucoma are presented in Table 4. Six participants responded that glaucoma was high eye pressure, and the remaining 2 responded that glaucoma was high eye pressure causing blindness. The source of knowledge about glaucoma in 7 of 8 respondents who were aware of glaucoma was information from acquaintances who had history of glaucoma. The remaining one respondent who had lost vision in one eye got the information from an eye doctor in a hospital.

**Table 4:** Answers responded to various questions by those participants who were aware of glaucoma at an ophthalmic outreach site in southwestern Ethiopia, 2009.

Response	no (%) [N = 8]
**Meaning of glaucoma**	
High eye pressure	6 (75)
High eye pressure causing blindness	2 (25)
**Source of information**	
Close acquaintances	7(87.5)
Ophthalmologist	1(12.5)
**Can someone have glaucoma without any symptoms?**	
Yes	1 (12.5)
No	3 (37.5)
Not sure	4 (50.0)
**Is blindness due to glaucoma reversible?**	
Yes	1 (12.5)
No	1 (12.5)
Not sure	6 (75.0)
**Factors identified as risk for glaucoma**	
Family history	2 (25.0)
Old age	3 (37.5)
Not sure	3 (37.5)
**Perceived risk of getting glaucoma**	
High	1(12.5)
None	2 (25.0)
Not sure	5 (62.5)

One participant mentioned that one could have glaucoma without having any symptoms and the same patient believed that vision loss due to glaucoma was not reversible. Two participants believed that family history was a risk factor for developing glaucoma and another two identified old age as a risk factor. Only one participant perceived that he had risk of getting glaucoma himself. Five of the 8 respondents were not sure of their risk of getting glaucoma, and the remaining two responded none (Table 3). Only one of the respondents who were aware of glaucoma had personal history of glaucoma while the remaining 7 had not been previously screened for glaucoma or other eye conditions. All of the study participants who were aware of glaucoma had no history of use of prescription glasses, eye surgery, diabetes, hypertension or history of glaucoma or blindness in a first degree family.

## Discussion

Although a previous study conducted on glaucoma knowledge in a developing country defined it just as a 'yes' answer to the question 'do you know glaucoma?' [6], we declined to do so because we observed in the pretest procedure that most participants who said they heard about 'glaucoma' were really meant 'trachoma'. There has been a massive health education on trachoma through the national radio; and the awareness of the public about trachoma seems to have fairly increased though the confusion between trachoma and glaucoma appears to persist. This may be due to the fact that these two conditions have similar suffix and or the failure to discuss the difference between them in health education. There is no widely used term for either glaucoma or trachoma in any of the languages spoken in this study area or in Ethiopia at large. Therefore, we defined that a participant was aware of glaucoma if s/he said 'yes' to the question 'have you heard about glaucoma?' and s/he replied something like 'glaucoma is high eye pressure', 'glaucoma is high eye pressure causing blindness', 'glaucoma is high eye pressure causing damage to the eye nerve' or similar responses when asked 'what do you understand by glaucoma?'.

Our finding of 2.4% (8/340) of people aware of glaucoma among those attending outreach ophthalmic services in Southwestern Ethiopia appears to be similar to that found in urban (2.3%) [6], while higher than that found in rural (0.33%) [7] Southern India, respectively. It is noteworthy that these Indian studies were population-based epidemiologic surveys thus direct comparison may not be applicable.

Some studies from developed countries found relationship between male gender and lack of glaucoma awareness [8-10], while similar studies from a less developed country reported the reverse [6,7]. Whereas several other studies found no difference between male and female regarding awareness of glaucoma [11-14]. We did not also find an association between glaucoma awareness and gender.

The relationship between older age and awareness of glaucoma was found in previous reports [6,9,10]. In our study there was a trend towards the reverse though the difference did not reach statistical significance.

The only demographic factor associated with awareness of glaucoma in the present study was higher educational level. Similar finding was reported from a developing country [6,7].

Several studies reported that there was a strong association between glaucoma awareness and having family history of glaucoma [8,9,11,12,15]. Individuals having a family member or acquaintance with glaucoma may be provoked to search for more information. In addition glaucoma patients may contact family members or family members may bring the patients in for their eye appointments and gather the information from their eye doctor. Surprisingly, having personal history of glaucoma was not significantly associated with increased glaucoma awareness in a clinic-based study in the US [11].

Apart from low educational level, our survey did not identify other attributes associated with glaucoma unawareness. This could probably be because of low proportion of participants who were aware of glaucoma. Therefore, based on the results of our study, no particular group of people is assumed to be aware of glaucoma in this part of the country thus health education must be targeted to at least all individuals at risk of developing the disease regardless of gender, ethnicity, religion or occupation.

Regarding the source of information about glaucoma, friends were reported to be the main source in a German survey [13], while mass media were the main source in rural India [7]. Information from close acquaintances was apparently the only source in our study. We suppose that glaucoma patients who present to eye clinics may be an important means to disseminate information about glaucoma. Therefore, encouraging known glaucoma patients to prompt their family members undergo screening can be a good starting point leading to the detection of glaucoma in early stages.

Mass media as a source of information on glaucoma was not reported by any of the participants in this study. It may be due to two reasons. Firstly, many people may have limited access to the media; secondly, health education activities on the mass media are probably scanty. We suspect the later being the main reason. Recently transmission of a community radio service was started for the public in and around Jimma Zone. We believe that this opportunity can be utilized to communicate health education regarding glaucoma and other causes of avoidable blindness.

It is disturbing that nearly all of the respondents who were aware of glaucoma had not ever undergone screening. This indicates that having awareness about a disease is not sufficient to lead someone to put the knowledge into an appropriate practice. There may be other barriers which hinder people from seeking screening for glaucoma which need to be assessed with other more comprehensive studies.

Educating patients with glaucoma directly by a physician may not be feasible in Ethiopia due to low number of ophthalmologists for a disproportionately large number of patients. It can be done by ophthalmic nurses or ophthalmic medical assistants at the physician office under the monitoring of an ophthalmologist. Although educating a gathering of patients and companions at a waiting area of an eye unit may not be as effective as individual patient education, we believe that regular sessions by the former method is the most feasible and cost-effective means available at the moment in Ethiopia. It is obvious that health education sessions have to be conducted in a language each target individual understands.

Do we have the resources to screen many people for glaucoma is another big issue even if we would be able to increase glaucoma awareness and prompt many people demand screening. Free screening services may have to be availed for family members of glaucoma patients as it is done elsewhere [16,17].

Finally, though the participants of this study differ from the general population from which they came in that the former presented to seek treatment for an eye condition, almost none of the respondents who were aware of glaucoma got this information from an eye camp or a health facility. Therefore, similar results are likely to be duplicated in a population-based sample.

Future studies in other districts may give an overall picture of the awareness of glaucoma in this region of Ethiopia.

## Conclusion

We documented that awareness of glaucoma is very low among people attending ophthalmic outreach services in Southwestern Ethiopia. The level of knowledge and practice among those reporting to be aware of glaucoma was also poor. One can suspect that the level of glaucoma awareness will be similarly low in the general population.

An efficient information, education and communication (IEC) strategy needs to be designed to increase awareness of the community about glaucoma so that early diagnosis and treatment of individuals with this condition may be possible, thereby preventing needless visual impairment and preserving quality of life.

## Competing interests

The authors declare that they have no competing interests.

## Authors' contributions

AT conceived and designed the study, and coordinated and monitored data collection. BS contributed in the study design. AT performed analysis of the data. AD contributed in the statistical analyses. AT drafted the manuscript. BS and AD contributed to the review. All authors read and approved the final manuscript.

## Pre-publication history

The pre-publication history for this paper can be accessed here:

http://www.biomedcentral.com/1471-2415/10/17/prepub
